# Metabolomic Analysis of *Elymus sibiricus* Exposed to UV-B Radiation Stress

**DOI:** 10.3390/molecules29215133

**Published:** 2024-10-30

**Authors:** Fei Zhang, Ming Sun, Daxu Li, Minghong You, Jiajun Yan, Shiqie Bai

**Affiliations:** 1 College of Life Sciences and Engineering, Southwest University of Science and Technology, Mianyang 621010, China; 2 Sichuan Provincial Forestry and Glassland Key Laboratory of Innovation and Utilization of Grasses in the Tibetan Plateau, Sichuan Academy of Grassland Sciences, Chengdu 611731, China

**Keywords:** *Elymus sibiricus*, metabolomics, UV-B radiation stress, allantoin

## Abstract

Plants cultivated on the Qinghai-Tibet Plateau (QTP) are exposed to high ultraviolet radiation intensities, so they require effective mechanisms to adapt to these stress conditions. UV-B radiation is an abiotic stress factor that affects plant growth, development, and environmental adaptation. *Elymus sibiricus* is a common species in the alpine meadows of the QTP, with high-stress resistance, large biomass, and high nutritional value. This species plays an important role in establishing artificial grasslands and improving degraded grasslands. In this study, UV-B radiation-tolerant and UV-B radiation-sensitive *E. sibiricus* genotypes were subjected to simulated short-term (5 days, 10 days) and long-term (15 days, 20 days) UV-B radiation stress and the metabolite profiles evaluated to explore the mechanism underlying UV-B radiation resistance in *E. sibiricus*. A total of 699 metabolites were identified, including 11 primary metabolites such as lipids and lipid-like molecules, phenylpropanoids and polyketides, organic acids and their derivatives, and organic oxygen compounds. Principal component analysis distinctly clustered the samples according to the cultivar, indicating that the two genotypes exhibit distinct response mechanisms to UV-B radiation stress. The results showed that 14 metabolites, including linoleic acid, LPC 18:2, xanthosine, and 23 metabolites, including 2-one heptamethoxyflavone, glycyrrhizin, and caffeic acid were differentially expressed under short-term and long-term UV-B radiation stress, respectively. Therefore, these compounds are potential biomarkers for evaluating *E. sibiricus* response to UV-B radiation stress. Allantoin specific and consistent expression was up-regulated in the UV-B radiation-tolerant genotype, thereby it can be used to identify varieties resistant to UV-B radiation. Different metabolic profiles and UV-B radiation response mechanisms were observed between the UV-B radiation-tolerant and UV-B radiation-sensitive *E. sibiricus* genotypes. A model for the metabolic pathways and metabolic profiles was constructed for the two genotypes. This metabolomic study on the *E. sibiricus* response to UV-B radiation stress provides a reference for the breeding of new UV-B radiation-tolerant *E. sibiricus* cultivars.

## 1. Introduction

Solar ultraviolet radiation is divided into long-wave ultraviolet A (Ultraviolet-A, UV-A, 315–400 mm), medium-wave ultraviolet B (Ultraviolet-B, UV-B, 280–315 mm), and short-wave ultraviolet C (Ultraviolet-C, UV-C, 100–280 mm) according to the wavelength. UV-A has minimal effects on plant growth. Conversely, UV-C significantly affects plant growth and development by inducing changes in the structure of biomolecules such as DNA and proteins. However, UV-C in sunlight can be effectively absorbed by the ozone layer [1]. UV-B radiation is an abiotic stress factor that impacts plant growth, development, and environmental adaptation. High-intensity, continuous full-wavelength UV-B radiation is referred to as UV-B stress, and it causes abnormal plant growth and development [2]. UV-B radiation is an environmental stress factor that modulates various aspects of photomorphogenesis, such as the inhibition of hypocotyl elongation, cotyledon expansion, and flavonoid contents, and it induces physiological stress responses [3]. Sustained high-intensity UV-B radiation can directly cause DNA damage, accumulation of reactive oxygen species, lipid and protein oxidation, and disrupt photosynthesis [4], resulting in plant morphology changes such as leaf curling, increased leaf thickness, shortened internodes, root shoot ratio changes, wilting and yellowing. Plants develop various protective coping strategies, such as morphological changes, accumulation of secondary metabolites and endogenous macromolecules, and detoxification of reactive oxygen species, to alleviate the damage caused by UV-B radiation [5,6]. These response mechanisms enhance plant adaptation to high ultraviolet intensities and promote plant growth and development.

UV-B radiation stress causes complex changes in the genetic, physiological, biochemical, and phenotypic traits of plants. Plants develop various protective mechanisms to alleviate UV-B damage by switching from a growth state to a UV-B stress adaptation state [7]. The amount of UV-B radiation reaching the Earth’s surface depends on latitude, season, time of day, cloud cover, and altitude [8]. Natural UV-B radiation levels regulate gene expression, plant growth, and development [9]. However, high UV-B levels adversely affect cellular/subcellular and macromolecular molecules and processes in plant tissues, including DNA, RNA and protein damage, photosynthesis, biomass, and seed production, ultimately modulating plant structure [10]. Plants respond to high levels of UV-B radiation by inducing the expression of flavonoid biosynthesis genes, hence accumulating UV-absorbing flavonoid compounds [11]. For example, the *MYB4R1* gene in *Tartary buckwheat* is a key regulator of UV-B stress adaptation, primarily through up-regulating the synthesis of flavonoids and anthocyanins under UV-B stress conditions by binding to L-box motifs in *FtCHS*, *FtFLS*, and *FtUFGT* promoters [2]. BRI1-EMS-SUPPRESSOR 1 (BES1) is a transcription factor in *Arabidopsis thaliana* that binds directly to the promoter of these *MYB*s in a BR-enhanced manner, down-regulating the expression of *MYB11*, *MYB12*, and *MYB111* implicated in flavonol biosynthesis, ultimately reducing flavonol content. High-intensity UV-B radiation downregulates *BES1* expression, consequently promoting flavonol synthesis [12]. The UV resistance locus 8 (UVR 8) is a UV-B photoreceptor necessary for UV-B reaction, which regulates UV-B photomorphogenesis and UV-B stress tolerance [13,14]. This receptor modulates morphology and initiates plant defense mechanisms such as increased antioxidant molecules, light repair, and UV-B screening pigment accumulation [15]. UVR8 regulates transcription factors, such as MYB domain protein 73/77 (MYB73/MYB77), MYB domain protein 13 (MYB13), WRKY DNA-binding protein 36 (WRKY36), BES1-interacting MYC-like 1 (BIM1), and phytochrome interacting factor 4 (PIF4) and PIF5, to directly modulate transcription and photomorphogenesis [16,17,18,19,20]. Moreover, UVR8 promotes flavonoid biosynthesis, especially anthocyanins and flavonols, causing significant accumulation under UV-B stress [21,22]. Plant metabolites, such as ascorbic acid, phenols and flavonoids, effectively protect plants from UV-B radiation damage [23,24]. Glycerol is UV-B stress biomarker in *Rhododendron chrysanthum Pall* [25]. UV-B radiation increases starch synthesis, soluble protein content, and the activity of related metabolic enzymes [26,27,28]. Endogenous melatonin [29], kaempferol [12], phenylpropanoid, caprylic acid [7], and hydroxycinnamic acid modulate *Arabidopsis thaliana* L., *Astragali radix* [30], and *Dendrobii caulis* [31] response to UV-B radiation stress. In addition, plants counteract the adverse effects of UV-B radiation stress by accumulating osmotic substances such as proline and soluble sugars [32,33]. Moreover, these osmotic molecules absorb UV-B radiation in the epidermal cell layer, reduce UV-B transmission to the mesophyll layer, and thus protect the photosynthetic system. Furthermore, these molecules protect plant cells from the damage induced by reactive oxygen species and active nitrogen produced during stress response [9]. Several plants change the expression of genes and metabolites are implicated in processes, such as cellular responses, molecular changes, and metabolic regulation, to alleviate UV-B stress.

Plant resistance to UV-B radiation is a complex, involving many physiological and molecular processes, including changes in gene expression and cell metabolism. Advances in omics techniques, such as transcriptomics, proteomics, and metabolomics, have significantly improved the study of biological molecular mechanisms underlying UV-B radiation stress response. Metabolomics is an advanced technique used to study the changes in plants subjected to abiotic stress. This technique provides supplementary information for proteomics, transcriptomics, and genomics. Notably, this technique is an alternative strategy for stress-related studies, and it can be effectively performed on any species, regardless of whether they have a reference genome [34]. Metabolomics is widely used to study plant biotic and abiotic stress [35,36], especially in identifying biomarkers and analysis of plant phenotypes [37]. Non-targeted metabolomics (also known as discovery metabolomics) is primarily conducted through chromatography-mass spectrometry (LC-MS, GC-MS) to identify differentially expressed metabolites and then explore the relationship between metabolites and physiological or pathological changes [38]. This strategy is effective for detecting complex metabolites and their roles in animals and plants.

*Elymus sibiricus* (Siberian wildrye) is a model species for the *Poaceae* family in the *Elymus* genus. This species is a perennial, cold-season, self-pollinating high-quality forage plant. It is widely used in artificial grassland planting and ecological restoration of degraded grasslands in the QTP and alpine grassland regions at altitudes ranging from 2500 m to 4500 m due to its high-stress resistance, high biomass, easy cultivation, high crude protein content, and excellent adaptation to high-altitude conditions characterized by strong ultraviolet radiation [39]. The great adaptation to the UV-B radiation areas makes *E. sibiricus* an excellent research object to explore UV-B radiation tolerance mechanisms. However, studies on the UV-B radiation tolerance and mechanisms of *E. sibiricus* are limited due to lack of reference genomic information for *E. sibiricus*. In this study, the non-targeted metabolomic technique was used to explore the response of two *E. sibiricus* genotypes to UV-B radiation stress. The purpose of this study was to (i) identify the UV-B radiation-tolerant *E. sibiricus* genotype to provide germplasm resources and a basis for the breeding of UV-B radiation-tolerant varieties; (ii) identify the metabolites in *E. sibiricus* associated with response to UV-B radiation; (iii) compare the metabolite profiles of the two *E. sibiricus* genotypes under long-term and short-term UV-B radiation stress; (iv) explore the metabolic patterns of two *E. sibiricus* genotypes and identify key metabolic pathways associated with response to UV-B radiation stress. A metabolic model for predicting potential metabolic pathways associated with response to UV-B radiation stress was constructed.

## 2. Results

### 2.1. Screening of UV-B Radiation-Tolerant and Sensitive Genotypes of E. sibiricus

**Figure 1:**
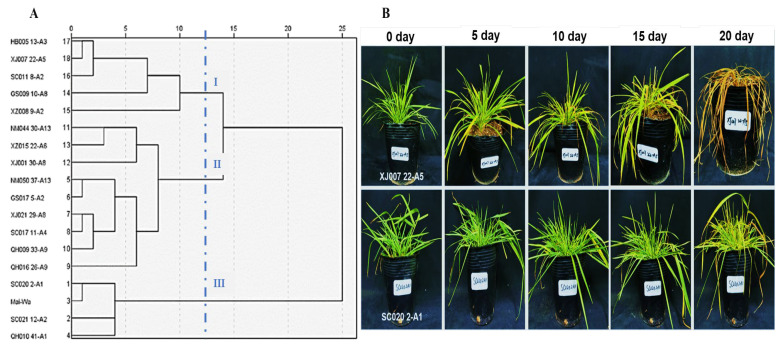
Screening of UV-B radiation-tolerant and sensitive genotypes of *E. sibiricus*. (**A**) Hierarchical clustering diagram of the 18 *E. sibiricus* samples; (**B**) phenotypic changes of the SC genotype and XJ genotype at days 0, 5, 10, 15, and 20 of UV-B radiation, respectively. SC represents the UV-B radiation-tolerant genotype and XJ represents the UV-B radiation-sensitive genotype.

A total of 18 samples of *E. sibiricus* were subjected to 288 KJ/m^2^ UV-B radiation intensity to evaluate their resistance to UV-B radiation. The 18 plant materials were grouped into three categories based on the hierarchical clustering results. Class I comprised the UV-B radiation-sensitive samples, Class II was the UV-B radiation moderately tolerant samples, and Class III comprised the UV-B radiation-tolerant samples (Figure 1A). Membership function analysis demonstrated that SC020 2-A1 and XJ007 22-A5 had the highest and lowest values (Table S1). The membership function analysis results were consistent with the hierarchical clustering results. Therefore, these two genotypes were designated the UV-B radiation-tolerant genotype (SC0202-A1, SC) and UV-B radiation-sensitive genotype (XJ007 22-A5, XJ) in the subsequent analyses. Under UV-B radiation stress, the two genotypes exhibited significant differences in morphological traits (Table S2). The leaves for the SC genotype had leaves that transitioned from emerald green to chlorotic to yellowing and ultimately wilted. The leaves of the XJ group changed from emerald green to chlorotic to yellowing, eventually withering and dying (Figure 1B). Phenotypic analysis further confirmed that the SC0202-A1 and XJ007 22-A5 genotypes were reliable for subsequent metabolomics studies as UV-B radiation-tolerant and UV-B radiation-sensitive genotypes, respectively.

### 2.2. Physiological Index Analysis of the Two Genotypes After UV-B Radiation Exposure

**Figure 2:**
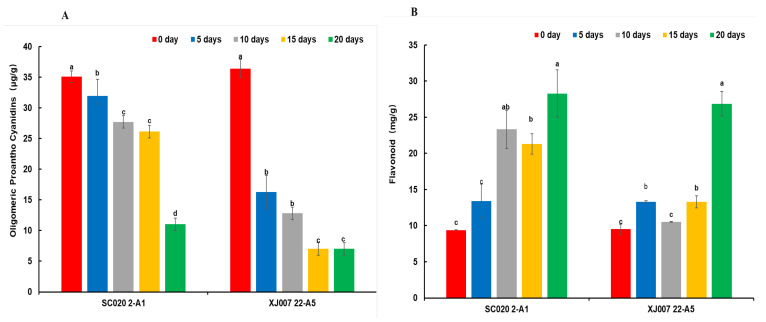
Physiological indicators of SC and XJ genotypes. (**A**) Proanthocyanidin content; (**B**) flavonoid content. Different lower-case letters indicate the significant difference at different UV-B radiation stress time for the same *E. sibiricus* variety at 0.05 levels. SC represents the UV-B radiation-tolerant genotype and XJ represents the UV-B radiatio-sensitive genotype.

The level of proanthocyanidins in the SC genotype and XJ genotype decreased with an increase in the duration of UV-B radiation exposure (Figure 2A). The content of proanthocyanidins in leaves of the two genotypes on day 20 was decreased by 69% and 81%, respectively, compared to day 0 (*p* < 0.05). The proanthocyanidin content of the SC genotype was higher compared to the XJ genotype at all time points except day 0. The levels of flavonoids in the SC and XJ genotypes increased with an increase in the duration of UV-B radiation exposure (Figure 2B). In the prophase of UV-B radiation exposure (5 and 10 days), the flavonoid level of the XJ genotype was significantly increased (*p* < 0.05), but there was no significant change in the SC genotype. At the later stages of UV-B radiation exposure (15 and 20 days), flavonoid levels were significantly elevated in both genotypes (*p* < 0.05). The relative water content and chlorophyll content of the two genotypes decreased with an increase in the duration of UV-B radiation exposure. The relative electrical conductivity increased with increasing UV-B radiation exposure time, especially in the XJ genotype, which significantly increased on day 20 (*p* < 0.05) (Figure S1A–C). The contents of osmoregulatory substances (proline and soluble protein) and antioxidant enzymes (peroxidase and superoxide dismutase) increased at varying degrees with prolonged stress time (Figure S1D–G).

### 2.3. Comprehensive Metabolite Profiling in E. sibiricus Grown Under Different UV-B Radiation Stress Times

Leaf samples from the two genotypes were collected after days 0, 5, 10, 15, and 20 of UV-B radiation exposure to explore the effects of UV-B radiation on the metabolite composition of *E. sibiricus*. Metabolite levels were determined by liquid chromatography-mass spectrometry (LC-MS). A total of 699 metabolites were identified in the 60 samples subjected to UV-B radiation stress (Table S3). The metabolites were further grouped into 11 classes, including 188 lipids and lipid-like molecules; 102 phenylpropanoids and polyketides; 62 organic acids and their derivatives; 53 oxygen-containing organic compounds; 44 organoheterocyclic compounds; 33 benzenoids; 31 nucleosides, nucleotides, and their analogs; eight lignans, neolignans and related compounds; six alkaloids and their derivatives; one nitrogen-containing organic compound, and 171 other metabolites (Figure 4A, Table S4). A total of 528 metabolites were annotated using the Kyoto Encyclopedia of Genes and Genomes (KEGG) compound database.

**Figure 3:**
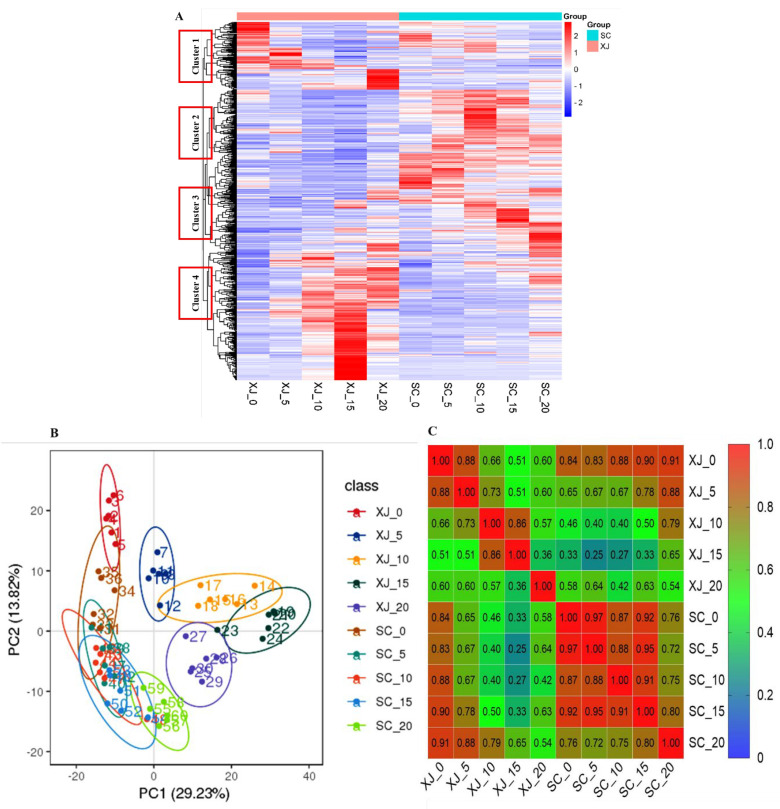
Metabolite profiles of the SC and XJ genotypes under different UV-B radiation stress times as determined by HCA and PCA. (**A**) Cluster analysis of the metabolites present in the SC and XJ genotypes. Red indicates high abundance, whereas blue indicates low abundance. Metabolites were clustered into four distinct clusters (group 1, group 2, group 3, group 4). (**B**) The PCA plot illustrating the metabolites in the different samples. Each point represents one metabolite profiling experiment, that is, the numbers 1 to 6 represent samples from day 0 of XJ radiation stress (XJ_0), 7 to 12 represent samples from 5 days of XJ radiation stress (XJ_5), 13 to 18 represent samples from 10 days of XJ radiation stress (XJ_10), 19 to 24 represent samples from 15 days of XJ radiation stress (XJ_15), 25 to 30 represent samples from 20 days of XJ radiation stress (XJ_20), similarly, numbers 31 to 60 represent samples of SC in different UV stress days (SC_0, SC_5, SC_10, SC_15, SC_20). Six biological replicates were set per UV-B radiation time point. (**C**) Pearson’s correlation heatmap of 60 samples. SC represents the UV-B radiation-tolerant genotype and XJ represents the UV-B radiation-sensitive genotype.

Multivariate statistical analyses were conducted to explore differentially expressed metabolites among the samples. Hierarchical clustering analysis (HCA) was performed to visualize global metabolite alterations in the two genotypes grown under different UV-B radiation time (Figure 3A). The samples were distinctively clustered into two groups based on the cultivated varieties, indicating a genotype-specific metabolite profile. The metabolites were grouped into four main clusters based on genotype-specific expression patterns. Cluster 1 and cluster 4 comprised metabolites that were primarily up-regulated under short-term and long-term radiation exposure to the XJ genotype. Cluster 2 and cluster 3 comprised metabolites that were up-regulated during the entire radiation exposure period for the SC genotype. This clustering indicated a genotype-specific effect on metabolite profile. Consequently, the number and type of metabolites up-regulated in cluster 4 can be used to approximate the UV-B radiation tolerance of different genotypes of *E. sibiricus* and evaluate whether *E. sibiricus* is subjected to UV-B radiation stress. Principal component analysis (PCA) was performed for all the metabolites to identify the key factors affecting the metabolite composition (Figure 3B). The first principle component (PC1, 29.23%) separated the XJ samples from the SC samples at days 5, 10, 15, and 20 of UV-B radiation exposure. Notably, the first and second principal components explained 43.05% of the total variance, clearly distinguishing the two genotypes of *E. sibiricus* (Figure 3B). These results indicated that the metabolite profiles of the two *E. sibiricus* genotypes changed under UV-B radiation stress. A heatmap was constructed based on Pearson’s correlation analysis of the sixty samples (Figure 3C). The correlation analysis showed a highly significant positive correlation among the six biological replicates. These results indicated that the method used precisely detected the metabolites in *E. sibiricus* subjected to UV-B radiation stress. Moreover, the high correlation implies that the experimental design and results are reliable for downstream analysis.

### 2.4. Identification of Multiple Groups of DAMs and Their Response to UV-B Stress

**Figure 4:**
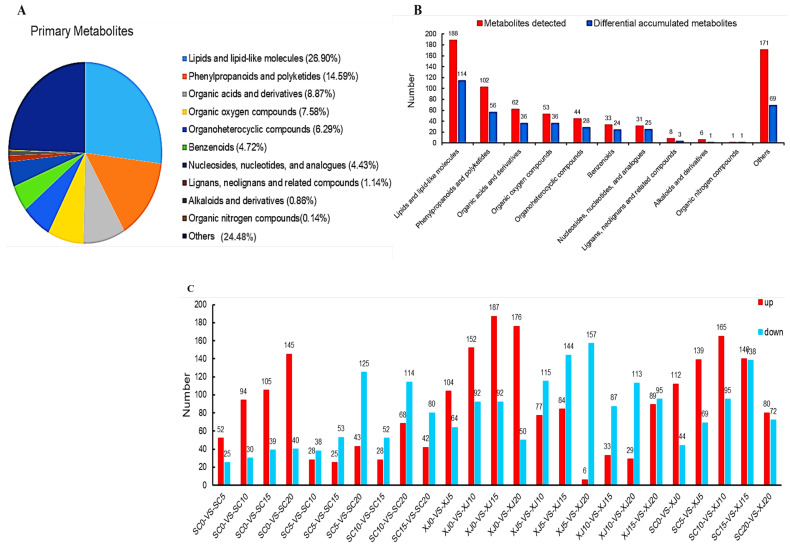
Metabolite classes and quantities. (**A**) Primary metabolite classes; (**B**) red represents all metabolite classes and their quantities, blue represents the quantities and classes of DAMs; (**C**) differentially expressed metabolites across the 25 comparison groups, up-regulated metabolites are shown in red and down-regulated metabolites are indicated in blue. SC represents the UV-B radiation-tolerant genotype and XJ represents the UV-B radiation-sensitive genotype.

The partial least square method (PLS-DA) was used to analyze 25 control groups to identify the metabolic response to UV-B radiation stress for the two genotypes. The variable important projection (VIP) value was used to determine differentially accumulated metabolites (DAMs), with metabolites with a VIP value ≥ 1 considered DAMs. A fold change threshold (FC) ≥2 or ≤0.5 indicated that the metabolite was up-regulated and down-regulated, respectively. A total of 393 DAMs were identified at days 0, 5, 10, 15, and 20 after UV-B radiation exposure (Figure 4B). The DAMs included 60.64% of the lipids and lipid-like molecules (114/188), 54.90% of the phenylpropanoids and polyketides (56/102), 58.06% of the organic acids and derivatives (36/63), 67.92% of organic compounds containing oxygen (36/53), 63.64% of organic heterocyclic compounds (28/44), 72.73% of benzenoids (24/33), 80.65% of nucleosides, nucleotides, and their analogues (25/31), 37.5% of lignans, neolignans and related compounds (3/8), 16.67% of alkaloids and their derivatives (1/6), 100% of the organic compounds containing nitrogen (1/1), and 40.35% of other metabolites (69/171). The amount of nitrogen-containing organic compounds (1/1, 100%) was not different before and after exposure to UV-B radiation stress, indicating that the expression of these substances is not induced by UV-B radiation stress. The results showed that 52, 94, 105, and 145 metabolites were up-regulated, whereas 25, 30, 39 and 40 metabolites were down-regulated in the SC genotype at days 5, 10, 15 and 20 of UV-B stress exposure compared with the control group. Conversely, 104, 152, 187, and 176 metabolites were up-regulated, whereas 64, 92, 92, and 50 metabolites were down-regulated in XJ genotype at days 5, 10, 15 and 20 after UV-B stress exposure compared to the control group (Figure 4C). Notably, the XJ genotype differentially expressed more metabolites compared to the SC genotype (Figure 4C), implying that under the same level of UV-B radiation stress injury, the XJ genotype needs to mobilize more DMAs to respond to UV-B radiation stress.

### 2.5. Effect of UV-B Stress on the Metabolite Profiles of the Two Genotypes

**Figure 5:**
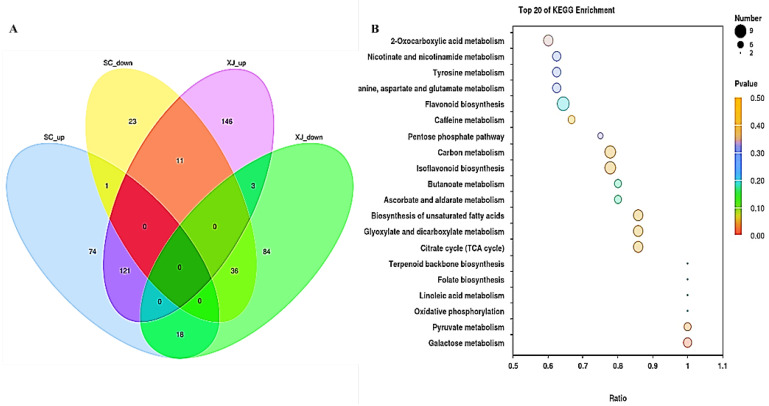
(**A**) A Venn diagram of the differentially expressed metabolites in the SC and XJ genotypes under UV-B radiation exposure; (**B**) significantly enriched KEGG pathways associated with the common DAMs between the two genotypes. SC represents the UV-B radiation-tolerant genotype and XJ represents the UV-B radiation-sensitive genotype.

A Venn diagram was constructed based on the up-regulated and down-regulated metabolites in SC and XJ genotypes to determine the number of DAMs in the 20 control groups of the two genotypes (Figure 5A). A total of 517 DAMs were identified in the 20 control groups after eliminating duplicates, with 284 DAMs in the SC genotype and 419 DAMs in the XJ genotype. Notably, 190 DAMs, including 121 up-regulated DAMs and 36 down-regulated DAMs, were common in the two genotypes. These shared metabolites are potential candidate metabolites to evaluate response to UV-B radiation stress. KEGG functional enrichment analysis of the 157 common DAMs under UV-B radiation stress demonstrated that they were mainly associated with significant enrichment of 2-oxy-carboxylic acid metabolism, nicotinate and niacinamide metabolism, tyrosine metabolism, threonine, aspartic acid and glutamate metabolism, flavonoid biosynthesis, caffeine metabolism, pentose phosphate pathway, carbon metabolism, isoflavone biosynthesis, butanoate metabolism, ascorbate and aldarate metabolism, unsaturated fatty acid biosynthesis, glyoxylate and dicarboxylic metabolism, citric acid cycle (TCA cycle), terpenoid synthesis, folate biosynthesis, and linoleic acid metabolism pathways (Figure 5B).

### 2.6. Metabolic Profiles of the Two E. sibiricus Genotypes Under UV-B Radiation Exposure

**Figure 6:**
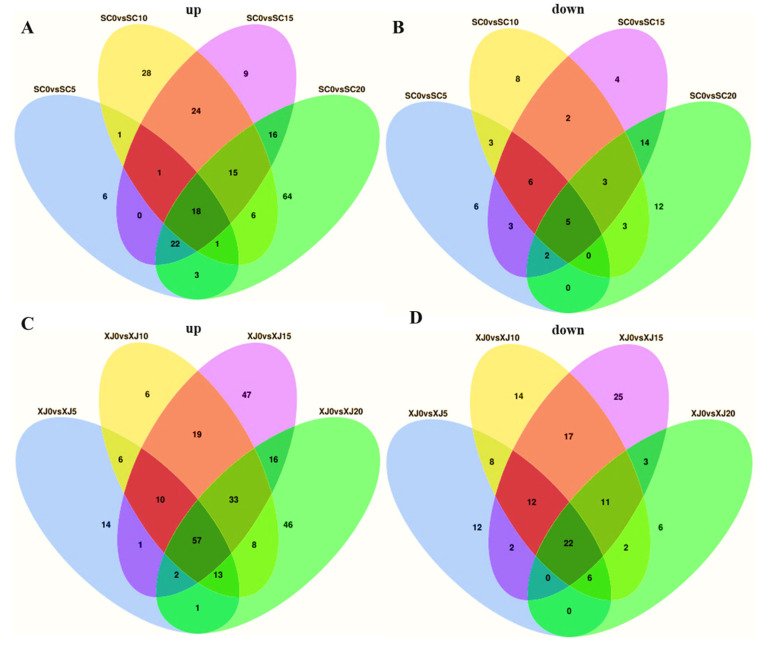

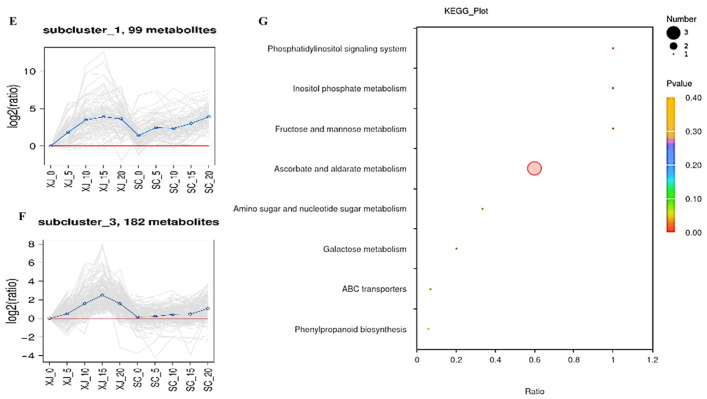
Metabolic profiles of the SC and XJ genotypes. A Venn diagram showing the number of up-regulated metabolites for the SC genotype (**A**) and XJ genotype (**C**). The number of down-regulated metabolites for the SC genotype (**B**) and XJ genotype (**D**). (**E**,**F**) Trend analysis of DAMs in the SC and XJ genotypes; (**G**) significantly enriched KEGG pathways associated with the DAMs unique to the SC genotype. SC represents the UV-B radiation-tolerant genotype and XJ represents the UV-B radiation-sensitive genotype.

A Venn diagram of DAMs was constructed to explore the metabolic profiles of the two genotypes under short-term and long-term UV-B radiation stress exposure. The results revealed that 18 metabolites were up-regulated and 5 were down-regulated in the SC genotype under short-term (5 days, 10 days) and long-term (15 days, 20 days) UV-B radiation exposure, respectively (Figure 6A,B). The XJ genotype had 57 up-regulated and 22 down-regulated metabolites under short-term (5 days, 10 days) and long-term (15 days, 20 days) UV-B radiation exposure, respectively (Figure 6C,D). The results showed that the accumulation of 23 metabolites in the SC genotype (Table S5) and 79 metabolites in the XJ genotype (Table S6) was mainly induced by UV-B radiation stress.

The findings indicated the contents of glycine anhydride, 2-aminoadipic acid, thymidine, adenosine, linoleic acid and LPC 18:2 in the SC genotype were significantly different, and the level of heptamethoxyflavone, corticosterone, isomucronulatol 7-o-glucoside, 5-hydroxytryptophan and pyrophosphate in the XJ genotype varied significantly under short-term UV-B radiation stress conditions (Table S7). These results indicate that these DAMs can be used as UV-B radiation stress biomarkers in *E. sibiricus* to evaluate the response of plants resistant to UV-B radiation and sensitive to UV-B radiation stress under short-term UV-B radiation exposure. Similarly, DAMs such as allantoin, ornithine, L-tryptophan, 5-aminovaleric acid, LPA 14:0, gluconolactone, vitamin C, danshensu, stachyose, daphnetin, (+)-dihydrojasmonic acid, paederoside, raffinose, glycyrrhizin, caffeic acid, were mainly expressed in the SC genotype under long-term UV-B radiation stress, and ethylmalonic acid, xanthosine, hypoxanthine-9-beta-D-arabinofuranoside, N-acetylvaline, 4-acetamidobutyric acid (Table S7) mainly expressed in the XJ genotype under long-term UV-B radiation exposure. These DAMs can serve as biomarkers for detecting UV-B radiation-tolerant and UV-B radiation-sensitive *E. sibiricus* genotypes exposed to long-term UV-B radiation stress. Interestingly, during the entire radiation exposure period, allantoin and geniposidic acid were up-regulated in the SC genotype. These results indicated that the up-regulation of allantoin and geniposidic acid can be used to determine the response of plants with UV-B radiation-tolerant genotype to UV-B radiation stress.

The differences in metabolite profiles of the two *E. sibiricus* genotypes were evaluated to determine the impacts of UV-B radiation stress on these genotypes. Four metabolite subclusters were identified (Figure S2). Subclusters 1 and 3 exhibited similar trends in SC and XJ genotypes (Figure 6E,F). Further analysis was conducted, and duplicates were discarded, resulting in 30 and 19 DAMs after subjecting the SC and XJ genotypes to long-term UV-B radiation stress (15 days and 20 days), respectively (Table S8). KEGG enrichment analysis was performed for the 30 DAMs in the SC genotype to explore the metabolic mechanisms underlying UV-B radiation tolerance of *E. sibiricus*. The results showed that these DAMS were associated with significant enrichment of ascorbate and aldarate metabolic pathways (Figure 6G).

### 2.7. UV-B Radiation-Tolerant and UV-B Radiation-Sensitive Genotypes Have Distinct Metabolic Pathways

**Figure 7:**
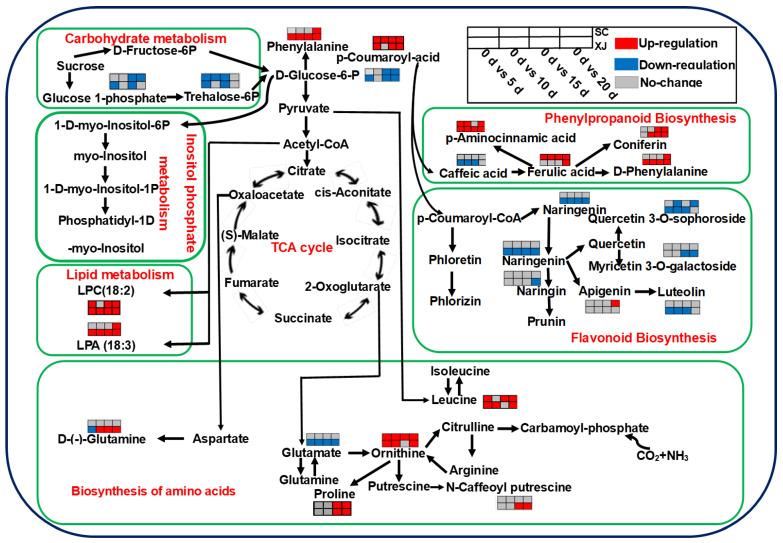
The metabolic network in *E. sibiricus* under UV-B radiation stress. The proposed metabolic pathways are based on a literature review and KEGG database analysis. Red indicates a significant up-regulation, blue indicates a significant down-regulation, and gray indicates no significant change in expression. SC represents the UV-B radiation-tolerant genotype and XJ represents the UV-B radiation-sensitive genotype.

A literature review and KEGG metabolic pathway analyses were conducted to explore the variation in metabolic profiles of the two *E. sibiricus* genotypes under UV-B radiation stress. The main pathways associated with UV-B radiation stress included the citric acid cycle (TCA cycle), phenylalanine biosynthesis, flavonoid biosynthesis, amino acid biosynthesis, phosphoinositide metabolism, lipid metabolism, carbon metabolism, and leucine, isoleucine, glutamate biosynthesis, and so on (Figure 7). Citric acid (TCA cycle), isocitrate (TCA cycle), alpha-ketoglutaric acid (TCA cycle), succinate (TCA cycle), D-(-)-glutamine (D-amino acid biosynthesis), leucine, isoleucine, ornithine and proline (amino acid biosynthesis and metabolism), coniferin (phenylalanine biosynthesis), and leaf protein LPC (18:2) (lipid metabolism) were up-regulated at irregular intervals under the four UV-B radiation intensities. On the contrary, naringenin (flavonoid biosynthesis), quercetin 3-O-sophoroside (flavonoid biosynthesis), quercetin 3-O-galactoside (flavonoid biosynthesis), naringin, luteolin (flavonoid biosynthesis), caffeic acid (phenylalanine biosynthesis), glucose 1-phosphate (carbon metabolism), and trehalose-6P (carbon metabolism) were down-regulated at irregular intervals under the four UV-B radiation time. These findings indicated that UV-B radiation stress effectively modulated the expression of these metabolites, ultimately regulating the related metabolic pathways. Malonic acid, p-amino-amino acid and ornithine were up-regulated in the two genotypes under short-term (5 days, 10 days) UV-B radiation. LPC, coniferin, and proline were up-regulated in the two genotypes under long-term (15 days and 20 days) UV-B radiation exposure. Interestingly, naringenin and glutamate exhibited similar expression profiles in both genotypes. This observation implies that the difference in UV-B radiation resistance can be partially attributed to the difference in the expression profiles of naringenin and glutamate in the two *E. sibiricus* genotypes.

## 3. Discussion

Understanding the mechanisms underlying UV-B radiation-tolerance is critical for improving global food security, owing to the growing population. UV-B radiation stress induces changes in metabolite profiles and the associated molecular pathways, ultimately enhancing the plant’s adaptability and resistance to stress. In this study, two genotypes with differences in resistance to UV-B radiation were selected from 18 *E. sibiricus* varieties from different habitats to explore the mechanism underlying resistance to UV-B radiation. The morphological, physiological, and metabolic changes were assessed after exposure of the two genotypes to UV-B radiation stress. The metabolite profiles of UV-B radiation-tolerant and UV-B radiation-sensitive *E. sibiricus* accessions exposed to UV-B radiation were evaluated by HPLC-MS. The results indicated that the UV-B radiation response of *E. sibiricus* is a complex process involving various metabolites involved in several pathways, including niacin and niacinamide metabolism, tyrosine metabolism, carbon metabolism, flavonoid biosynthesis, TCA cycle, alanine, aspartate and glutamate metabolism, inositol phosphate metabolism, lipid metabolism, folic acid biosynthesis, and oxidative phosphorylation. In this study, 30 differentially expressed metabolites were identified between the UV-B radiation sensitive and UV-B radiation tolerant accessions. These metabolites are potential markers to evaluate UV-B radiation tolerance in *E. sibiricus* and related species.

UV-B radiation damage to plants is primarily due to the denaturation of nucleic acids, proteins, and other macromolecules, resulting in the disruption of the membrane structure, membrane permeability changes, cell autolysis, and reduction in chlorophyll content [40]. Leaf damage features, including dead spots, curling, greening, and wilting, are the most intuitive parameters for evaluating stress in plants. Plant growth decreases with an increase in stress time. In addition, the electrical conductivity increases and the relative water content and chlorophyll content of leaves decrease significantly with an increase in stress duration. These changes decrease the photosynthetic efficiency, ultimately affecting biological processes such as growth, development and secondary metabolism [4,41,42].

Osmoregulation is a process of maintaining a balance of water and solutes in organisms, including plants, which occurs under various stress conditions, such as UV-B radiation stress. Solutes actively accumulate in the cell to reduce the osmotic potential of the cytoplasm and prevent excessive water loss [43]. Carbohydrates, amino acids, and soluble proteins are key metabolites and signaling intermediates in plant response to UV-B radiation stress [30]. In this study, the contents of soluble sugars, including turanose and sucrose, were significantly higher in the UV-B radiation-tolerant genotype than in the UV-B radiation-sensitive plants under control treatment. These soluble sugars may increase the osmotic potential and provide an adaptive buffer for UV-B radiation-resistant *E. sibiricus*. However, the level of soluble reducing sugar content with increasing stress time, which may be attributed to the demand for energy metabolism in the later stress stages.

Proline is an amino acid and osmoregulatory metabolite. Proline expression changes in response to UV-B radiation stress. Under stress, it is mainly localized in the subcellular structure and it also detoxifies reactive oxygen species [44]. The proline contents were significantly higher in the UV-B radiation-tolerant and UV-B radiation-sensitive genotypes than in the control group throughout the study period. Accumulation of proline requires a large amount of glutamate supply because it is synthesized from this amino acid [45]. Consequently, the glutamate content in the UV-B radiation-tolerant genotype decreased significantly under long-term UV-B radiation stress. This observation can be attributed to the consumption of high glutamate amounts to synthesize proline in response to UV-B radiation stress. This finding indicates that modulation of proline content is an important strategy for UV-B-tolerant genotypes to respond to UV-B radiation stress. The soluble protein content significantly increased in the two genotypes in the last phase of UV-B radiation stress exposure. The expression of linear predictive coding (LPC) and lipoprotein(a) was significantly up-regulated in the UV-B radiation-sensitive genotype during the entire treatment period. Soluble proteins induced by UV-B radiation stress enhance the cell membrane integrity. Plants maintain the carbohydrate biosynthesis pathway without disruption through the TCA cycle. The intermediate products of the TCA cycle, such as citrate, α-ketoglutarate and succinate, exhibited varying contents UV-B radiation stress [46]. In this study, citrate content was significantly high in both genotypes under UV-B radiation stress. Therefore, citrate content can be used as a marker in plants exposed to UV-B radiation stress. Exogenous application of citrate enhances the plant tolerance to abiotic stress, making it an effective method for enhancing UV-B radiation resistance in plants [47]. The findings indicate that these metabolites are essential for the adaptation of *E. sibiricus* to UV-B radiation stress.

Plants prevent and limit fatal damage from UV-B radiation by inducing antioxidant defense mechanisms and increasing flavonoid content. Several factors involved in plant UV-B response have been identified in mutant *Arabidopsis thaliana*. The findings indicated defective biosynthesis of ascorbate, flavonoids, and hydroxycinnamic acid in UV-B radiation-sensitive plants [4,48]. Some genes implicated in flavonoid biosynthesis are up-regulated, and flavonoid content increases in plants subjected to UV-B radiation stress [49,50]. Under UV-B radiation stress, proanthocyanidins are degraded to produce C-4 hydroxyl intermediates, ultimately forming ketones and aldehydes [51]. These findings explain the gradual decrease in the level of proanthocyanidins and the increase in flavonoids in the two genotypes explored in this study. Under UV-B radiation stress, the UV-B radiation-sensitive genotype exhibited higher contents of metabolites implicated in response to UV-B radiation stress compared to the UV-B radiation-tolerant genotype. This difference may be because the UV-B radiation-sensitive genotype had more sensitive stress receptors, resulting in more severe damage under UV-B radiation stress [52].

The UV-B radiation time can be as required for indoor plants. Conversely, the time of exposure to UV-B radiation for naturally growing plants in the field is not fixed. Therefore, it is imperative to explore the adaptative mechanisms in plants subjected to short-term and long-term UV-B radiation stress. In this study, the metabolite profiles of the two *E. sibiricus* genotypes were evaluated under short-term and long-term UV-B radiation stress. The findings revealed that DAMs had different metabolite profiles under the two conditions. The plant metabolic response to UV-B radiation stress is a dynamic equilibrium process and metabolism level increases to adapt to long-term UV-B radiation exposure.

The profiles of DAMs in the UV-B radiation tolerant and UV-B radiation sensitive genotypes subjected to short-term UV-B radiation response were different. The expression of glycine anhydride, 2-aminoadipic acid, and maleamic acid were modulated in the UV-B radiation tolerant genotype subjected to short-term radiation stress. Conversely, the contents of ethylmalonic acid, and D-arabinofuranoside were different in the UV-B radiation-sensitive genotype exposed to short-term UV-B radiation stress. These findings indicate that the genotypes exhibit complex and varying metabolite expression under short-term UV-B radiation, which is consistent with the proteomic results on *Triticum aestivum* [53] and transcriptomic and metabolomic findings on *pakchoi* [54]. The two genotypes exhibited a higher number of up-regulated or down-regulated DAMs under long-term UV-B radiation stress compared to term short-term UV-B radiation stress. Notably, some DAMs such as glycine anhydride, maleamic acid, and ornithine exhibited identical profiles in the genotypes under long-term UV-B radiation stress. This finding indicates that the two genotypes have similar metabolite profiles under long-term UV-B radiation exposure. The DAMs in the UV-B radiation-tolerant genotype were significantly less compared to the UV-B radiation-sensitive genotype, primarily because the UV-B radiation-tolerant genotype effectively responded to UV-B radiation signals in the initial phase of UV-B radiation exposure. In addition, the tolerant genotype can rapidly respond to UV-B radiation stress by modulating gene expression [4,55,56]. The high tolerance of the UV-B radiation-tolerant genotypes is mainly attributed to these features. UV-B radiation-tolerant genotypes can easily adapt to long-term UV-B radiation stress by modulating gene expression and ensuring stable metabolism in the early exposure stages [57]. In the last stage of stress, energy metabolism is reduced by modulating the transcription, and more energy is used to respond to UV-B radiation stress [58]. However, although down-regulation of gene expression in early stages may have reduced energy expenditure in the UV-B radiation-sensitive genotype, this energy is used to regulate gene expression and to respond to UV-B radiation stress at advanced stages of UV-B radiation exposure. In summary, the two genotypes exhibited varying adaptation mechanisms when exposed to UV-B radiation stress. The UV-B radiation stress response mechanisms of the UV-B radiation-tolerant genotype enhanced adaptation to circumvent long-term UV-B radiation stress.

**Figure 8:**
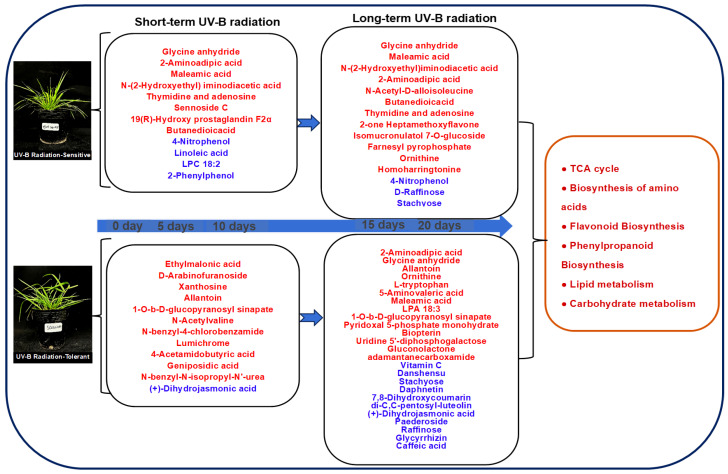
Metabolite profiles in the SC and XJ genotypes in response to UV-B radiation stress. The red font indicates increased expression, whereas the blue font indicates decreased expression. SC represents the UV-B radiation-tolerant genotype and XJ represents the UV-B radiation-sensitive genotype.

In this study, 30 metabolites were unique to the UV-B radiation-tolerant genotype. Among these metabolites, allantoin and geniposidic acid were significantly up-regulated, providing a mechanism for UV-B radiation-tolerant genotypes to alleviate UV-B radiation stress. Previous findings demonstrated that allantoin is an important metabolite for modulating response to UV radiation stress in tomato (*Solanum lycopersicum*) [59] and *Arabidopsis thaliana* [60], with higher contents observed under UV radiation stress. This metabolite promotes plant growth and is implicated in plant stress response, exogenous application of allantoin can enhance plant tolerance to adverse environments [61]. Therefore, allantoin is a potential metabolic biomarker for evaluating response to UV-B radiation stress in the UV-B radiation-tolerant genotype. Notably, the 30 DAMs were associated with significant enrichment of ascorbate and aldarate metabolic pathways. Ascorbate and aldarate metabolic pathways are involved in alleviating oxidative damage in plants and eliminating superoxide free radicals and reactive oxygen species produced by photosynthesis and UV-B radiation stress [62]. Consequently, the pathways regulate the redox potential in vivo, enhancing plant growth [63]. In this study, a simplified model was constructed based on the study results to describe the response of the UV-B radiation-tolerant and UV-B radiation-sensitive genotypes to short-term and long-term UV-B radiation at the metabolic level (Figure 8).

## 4. Materials and Methods

### 4.1. Plant Sample Collection and UV-B Radiation Treatment

Eighteen representative *E. sibiricus* materials were collected from Northeast, Northwest, North China, and QTP regions based on altitude and regional habitat differences (Table S9). Evenly plump *E. sibiricus* seeds were disinfected with 75% alcohol for 2 min and rinsed 3 times. Subsequently, the seeds were disinfected with 10% NaClO solution for 8 min and rinsed 5 times. The sterilized seeds were then placed in a Petri dish with a 10 cm diameter and covered with 3 layers of filter paper. The seedlings were transplanted into a pot when they reached the three-leaf stage. The 18 plant materials were transplanted into POTS (dried soil:nutrient soil:vermiculite = 3:4:1.5, diameter × height = 16 × 16 cm), and each material was planted in 20 POTS with one single plant in each pot. The plants were regularly watered after transplantation to ensure normal growth. UV-B radiation treatment was performed after 2 months of growth. The experiment was conducted in the central laboratory of Sichuan Provincial Grassland Science Research Institute (N 30°45′56″, E 103°57′53″). All the test materials were placed in the ultraviolet radiation lamp cabinet, with the temperature maintained at 20 °C, and the photocycle was set to 12 h of light and 12 h of darkness. UV-B fluorescent lamp tube was wrapped with a 0.125 mm thick cellulose diacetate film to filter UV-C and ensure the stability of light quality. The UV-B fluorescent lamp radiation irradiation was induced every day between 10:30 a.m.–14:30 p.m. The radiation intensity was 288 KJ/m^2^ (simulated plateau UV intensity of 120%, the intensity was referred to the radiation intensity of the QTP [64] and it was combined with the actual monitoring value of Hongyuan), and the rest of the time is normal light. The UV-B radiation intensity was measured at the top of the plant using an ultraviolet irradiation meter (Shanghai Gaozhi Precision Instrument Co., Ltd., Shanghai, China). Samples were taken at 0 (CK), 5, 10, 15 and 20 days of UV-B radiation. The treatment time points were selected because the maximum tolerance time was about 20 days during the pre-experiment. In addition, it was necessary to ensure that there were still old mang wheat alive under the maximum treatment time to meet the requirements of follow-up sampling. Secondly, obvious phenotypic differences were observed among different genotypes under different UV-B radiation stress time. Finally, based on the literature research report [65]. At each time point, 3 POTS with basically consistent growth from each material were selected as 3 replicates for sampling. Moreover, samples for metabolite analysis were also sampled for later determination. The sampling time was between 15:00–17:00 p.m. After sampling, the samples were immediately frozen in liquid nitrogen and then stored in a low-temperature refrigerator at −80 °C for subsequent determination of physiological indexes and metabolome. A total of 60 samples were collected (six biological replicates × two genotypes × five time points). Relative electrical conductivity, relative water content and chlorophyll content were determined based on fresh leaves.

### 4.2. Analysis of Phenotypic and Physiological Parameters

Anthocyanins were extracted using an acidic ethanol solution and the content was determined by single wavelength colorimetric method at an absorbance of 520 nm. The flavonoid content was determined using a kit (Suzhou Keming Biotechnology Co., Ltd., Suzhou, China). The samples were added to an alkaline nitrite solution. The flavonoid and aluminum ions formed a red complex with characteristic absorption peak at 510 nm. The absorption of the sample extraction solution was measured at 510 nm to determine the flavonoid content of the sample. The relative water content (RWC) was measured by the saturation weighing method. Fresh leaves (0.2 g) were weighed and the weight was recorded as fresh weight (FW). Subsequently, the leaves were soaked in distilled water for 12 h, wiped dry and the saturated constant weight (CW) was weighed. The samples were then placed in the oven at 80 °C and dried for 24 h and then weighed to obtain the dry weight (DW). The leaf relative water content (%) was calculated using the formula: (FW − DW)/(CW − DW) × 100%. The relative electrical conductivity (REC) was measured by the conductivity method. In brief, 0.1 g of fresh leaves were weighed and soaked in deionized water for 12 h. Subsequently, the electrical conductivity value S1 was measured with an electrical conductivity instrument. Further, the samples were placed in a 100 °C water bath for 30 min, and the S2 value was measured after cooling, as follows: REC% = S1/S2 × 100%. The activities of peroxidase and superoxide dismutase were determined with a kit. The activity of antioxidant oxidase was calculated by determining the light absorption rate at a specific wavelength. The chlorophyll content was determined using the SPAD-502 Plus portable chlorophyll analyzer. Five mature leaves were selected from each pot material and their average values were determined. The test results were expressed as a Spad index. Soluble protein and proline contents were determined according to the method reported by Tevini et al. [66]. The proline content was determined using the ninhydrin colorimetry of the kit. The soluble protein contents were determined by the Coomasil bright blue method (Suzhou Keming Biotechnology Co., Ltd., Suzhou, China). Phenotypic analysis was conducted using a method described by Tevini et al. [66], with minor modifications. The PS value was determined by the degree of leaf wilting, curling, and the number of yellowed leaves (Table S2).

### 4.3. Metabolite Extraction and UPLC-MS Sample Preparation

Tissues (100 mg) were grounded with liquid nitrogen, and the homogenate was resuspended in prechilled 80% methanol by vortexing. The samples were incubated on ice for 5 min and centrifuged at 15,000× *g* and 4 °C for 20 min. A portion of the supernatant was diluted to achieve a final concentration containing 53% methanol using LC-MS-grade water. The diluted samples were transferred to a fresh Eppendorf tube and centrifuged at 15,000× *g* and 4 °C for 20 min. The supernatant was then injected into the LC-MS/MS system [67].

### 4.4. Metabolite Detection by UHPLC-MS/MS

UHPLC-MS/MS analyses were performed using a Vanquish UHPLC system (Thermo Fisher, Dreieich, Germany) coupled with an Orbitrap Q Exactive^TM^HF-X mass spectrometer (Thermo Fisher, Dreieich, Germany). These analyses were conducted at Novogene Co., Ltd., (Beijing, China). Samples were loaded to a Hypersil Gold column (100 × 2.1 mm, 1.9 μm) using a 12-min linear gradient at a 0.2 mL/min flow rate. The mobile phase for the positive and negative polarity modes comprised eluent A (0.1% FA in Water) and eluent B (Methanol). The solvent gradient was set as follows: 2% B, 1.5 min; 2–85% B, 3 min; 85–100% B, 10 min; 100–200% B, 10.1 min; 2% B, 12 min. The Q Exactive^TM^ HF (Thermo Fisher) mass spectrometer was operated in positive/negative polarity mode with a spray voltage of 3.5 kV, a capillary temperature of 320 °C, sheath gas flow rate of 35 psi, auxiliary gas flow rate of 10 L/min, S-lens RF level of 60, and auxiliary gas heater temperature of 350 °C.

### 4.5. Data Processing and Metabolite Identification

The raw data files generated by the UHPLC-MS/MS system were processed using the Compound Discoverer 3.3 (CD3.3, ThermoFisher) for peak alignment, peak picking, and quantitation of each metabolite. The main parameters were set as follows: the peak area was corrected with the first QC, actual mass tolerance, 5 ppm; signal intensity tolerance, 30%; and minimum intensity. Subsequently, the peak intensities were normalized to the total spectral intensity. The normalized data was used to determine the molecular formula based on additive ions, molecular ion peaks and fragment ions. The peaks were then matched with peaks available in the mzCloud (https://www.mzcloud.org/, accessed on 20 July 2024), mzVault and MassList databases to obtain accurate qualitative and relative quantitative results. Statistical analyses were performed using the R statistical software (R version R-3.4.3), Python (Python 2.7.6 version) and CentOS (CentOS release 6.6). Data that were not normally distributed were standardized using the formula: sample raw quantitation value/(sum of sample metabolite quantitation value/sum of QC1 sample metabolite quantitation value) to obtain the relative peak areas. The compounds with CVs of relative peak areas in QC samples greater than 30% were discarded before metabolite identification and relative quantification.

### 4.6. Data Analysis

The metabolites were annotated using the KEGG database (https://www.genome.jp/kegg/pathway.html, accessed on 20 July 2024), HMDB database (https://hmdb.ca/metabolites, accessed on 20 July 2024) and LIPIDMaps database (http://www.lipidmaps.org/, accessed on 20 July 2024). The metabolites were subjected to hierarchical cluster analysis (HCA) using R software (R version R-3.2.3) [68] (www.r-project.org/, accessed on 20 July 2024). Principal components analysis (PCA) and partial least squares discriminant analysis (PLS-DA) were performed using the metaX program [69]. Univariate analysis (*t*-test) was conducted to compare the differences between the radiation-exposed and unexposed groups (*p*-value). The metabolites with VIP > 1 and *p*-value < 0.05 and fold change ≥ 2 or FC ≤ 0.5 were considered to as differentially expressed metabolites. Volcano plots were used to filter metabolites of interest based on the log2 (Fold Change) and −log10(*p*-value) of metabolites using the ggplot2 R package.

The data were normalized using z-scores of the intensity areas of differentially expressed metabolites, and heatmaps were generated using the Pheatmap R package (R version R-3.4.3). The correlation between differentially expressed metabolites was evaluated using the cor() function in R (method = Pearson). Statistical significance of the correlated differentially expressed metabolites was determined using the cor.mtest() function in the R program. *p*-value < 0.05 was considered statistically significant. Correlation plots were generated using the corrplot package in the R program. The functions of these metabolites and the related metabolic pathways were predicted using the KEGG database. The metabolic pathways associated with differentially expressed metabolites were identified using the KEGG database. A metabolic pathway was considered enriched when the ratio met the criterion x/n > y/N. A metabolic pathway was considered statistically significantly enriched when the *p*-value was <0.05.

## 5. Conclusions

UV-B radiation stress alters metabolite contents and induces differential expression of metabolites in two *E. sibiricus* genotypes. In this study, 699 metabolites were identified and classified into 11 categories. The findings indicated that flavonoids, amino acids and their derivatives, and lipid alcohols played significant roles in response to UV-B radiation stress. The results demonstrated that allantoin is a potential metabolic biomarker of UV-B radiation stress. In addition, its specificity and consistent up-regulation can be used to determine UV-B radiation tolerance in *E. sibiricus*. The ascorbate and aldarate metabolic pathways, flavonoid biosynthesis, and TCA cycle were significantly associated with response to UV-B radiation stress. This study reports biomarkers for UV-B radiation stress and key metabolic pathways associated with response to this stress, providing key insights into the breeding of UV-B radiation-resistant *E. sibiricus* genotypes.

## Data Availability

Data available on request due to restrictions, e.g., privacy or ethics.

## Supplementary Materials

The following supporting information can be downloaded at: https://www.mdpi.com/article/10.3390/molecules29215133/s1. Figure S1. Determination of physiological indicators of *E. sibiricus* under UV-B radiation stress. Figure S2. DAMs profiles of the UV-B radiation-tolerant (SC) and UV-B radiation-sensitive (XJ) genotypes. Table S1. Comprehensive evaluation and ranking of membership functions of 18 *E. sibiricus* samples. Table S2. Morphological profiles of UV-B tolerant (SC) and UV-B sensitive (XJ) *E. sibiricus* genotypes. Table S3. Relative quantitative values of metabolites in each sample and the QC samples. Table S4. Primary classes and number of metabolites. Table S5. Metabolites in the UV-B radiation tolerant genotype (SC) detected under long-term and short-term UV-B radiation stress. Table S6. Metabolites in the UV-B radiation sensitive genotype (XJ) detected under long-term and short-term UV-B radiation stress. Table S7. DMAs common to the UV-B radiation tolerant (SC) and UV-B radiation sensitive (XJ) genotypes under short-term (5 days and 10 days) and long-term (15 days and 20 days) UV-B radiation stress. Table S8. Thirty key metabolites in the UV-B radiation tolerant (SC) and UV-B radiation sensitive (XJ) genotypes. Table S9. Information on the *E. sibiricus* germplasm materials.
