# Supplementary material for: Metabolomic Analysis of Elymus sibiricus Exposed to UV-B Radiation Stress
Source: Molecules. 2024 Oct 30;29(21):5133. doi: 10.3390/molecules29215133 (PMC11548012; doi:10.3390/molecules29215133)
Supplement: Supplementary file 1 [file molecules-29-05133-s001.zip › molecules-3200901-supplementary.pdf]

molecules MDPI

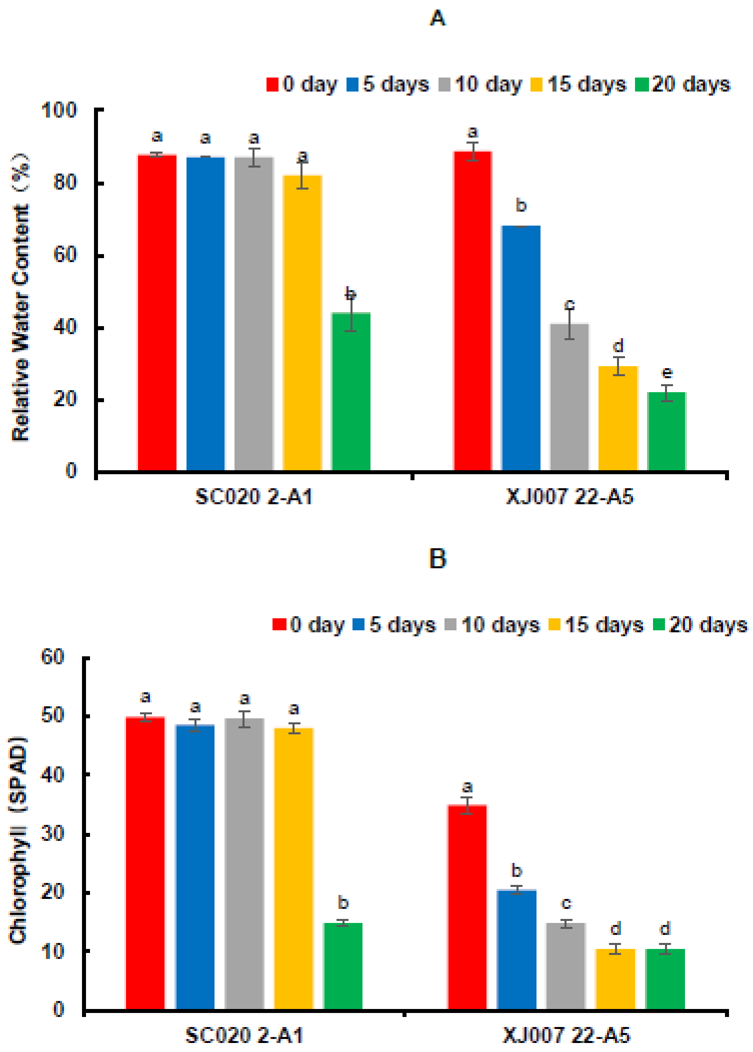

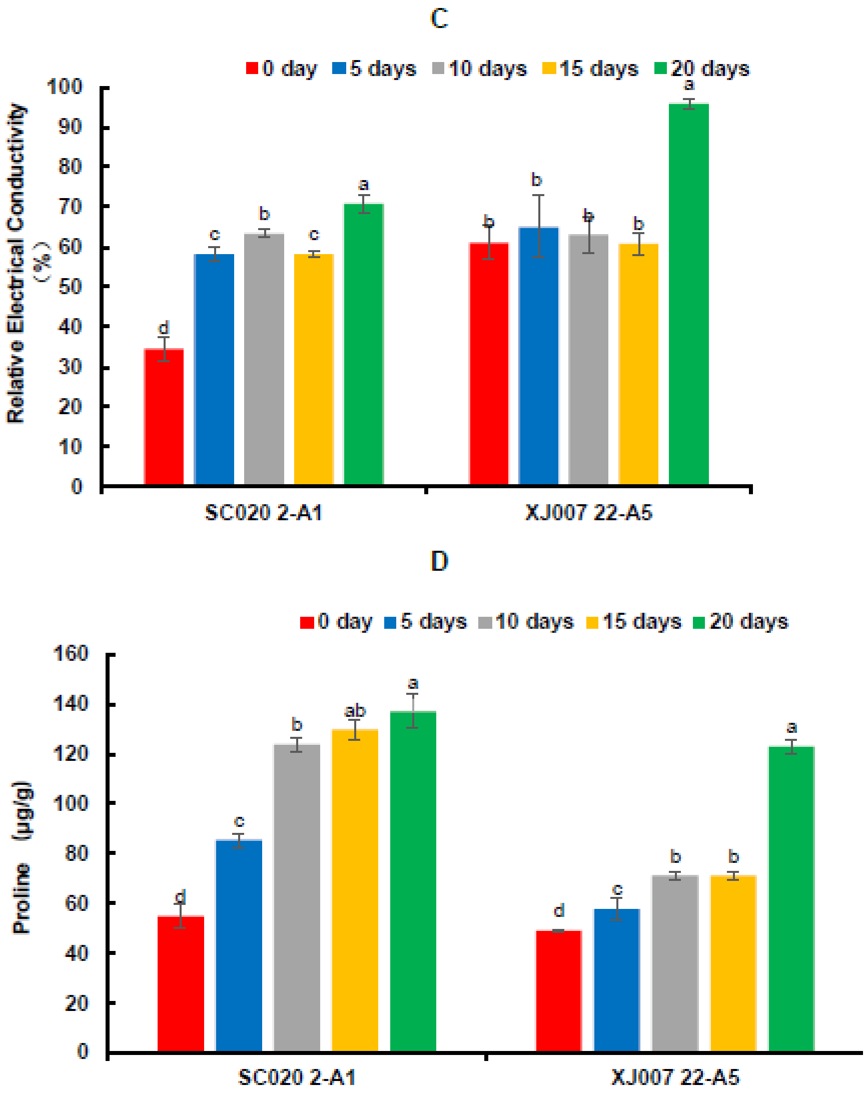
C
0 day
5 days
10 days
15 days
20 days
Relative Electrical Conductivity (%)
0
10
20
30
40
50
60
70
80
90
100
d
c
b
c
a
b
b
b
b
a
SC020 2-A1
XJ007 22-A5
D
0 day
5 days
10 days
15 days
20 days
Proline (μg/g)
0
20
40
60
80
100
120
140
160
d
c
b
ab
a
d
c
b
b
a
SC020 2-A1
XJ007 22-A5

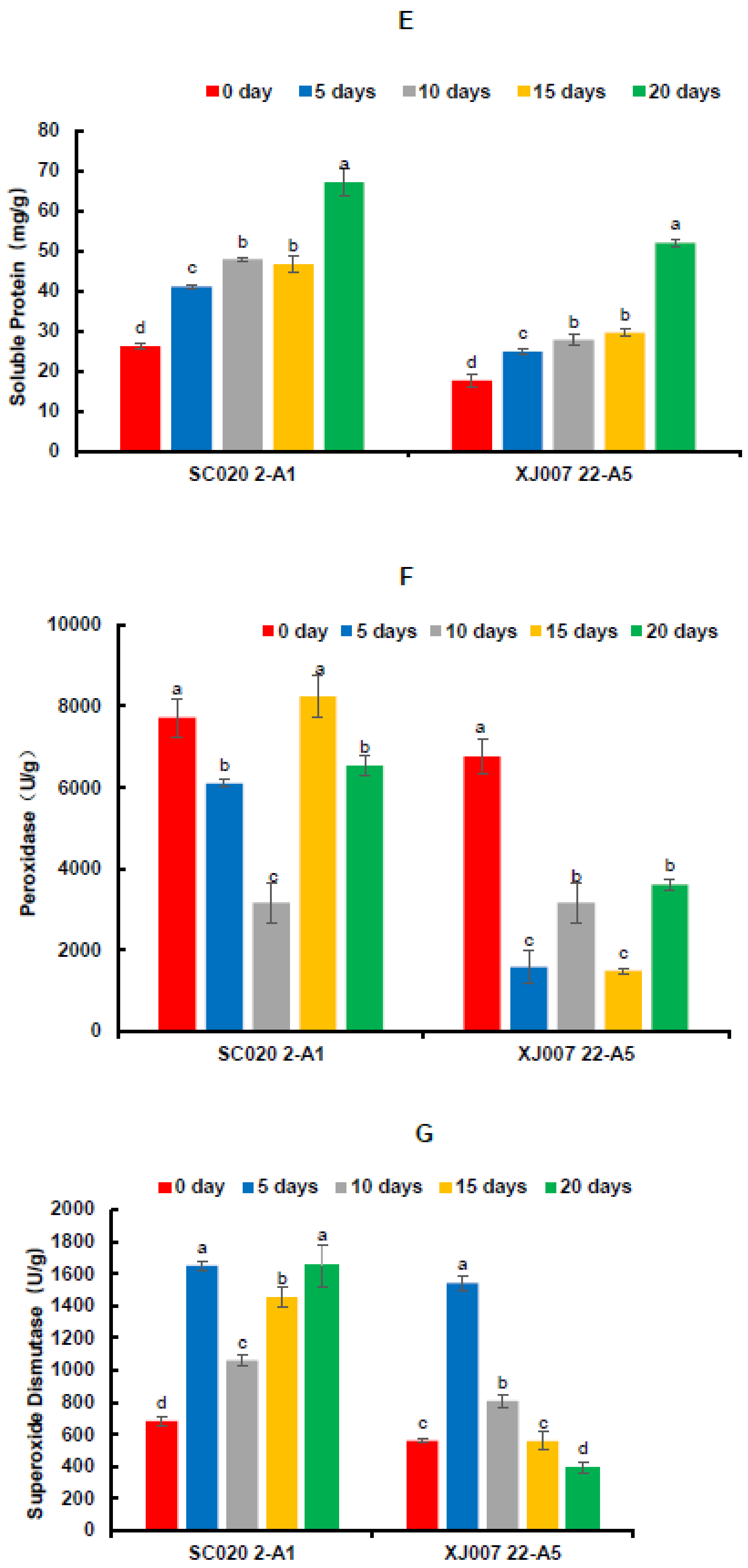

Figure S1. Determination of physiological indicators of *E. sibiricus* under UV-B radiation stress. (A) Relative water contents; (B) Chlorophyll contents; (C) Relative electrical conductivity; (D) Proline contents; (E) Soluble protein contents; (F) Peroxidase contents; (G) Superoxide dismutase contents.

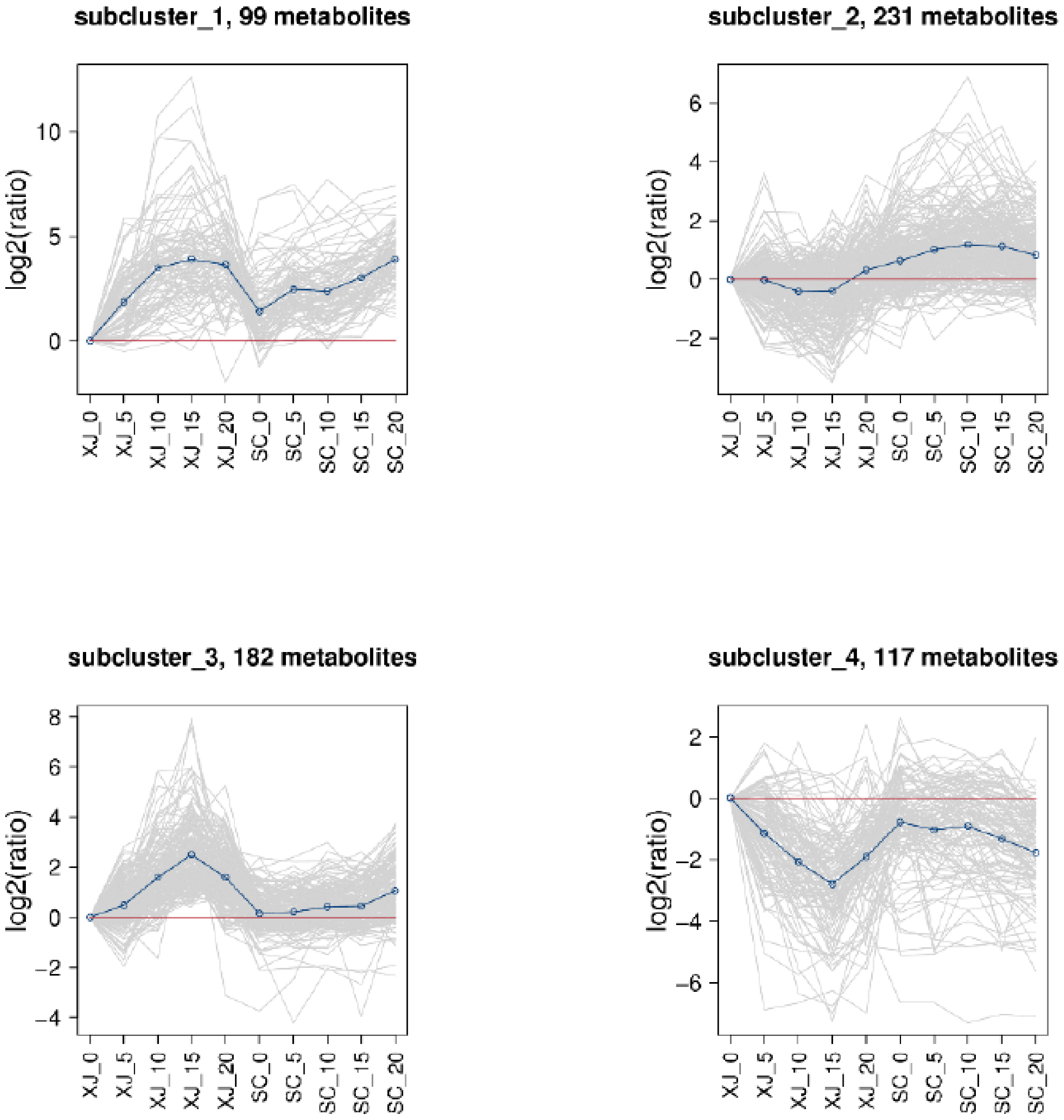

Figure S2. DAMs profiles of the UV-B radiation-tolerant (SC) and UV-B radiation-sensitive (XJ) genotypes.

Table S1. Comprehensive evaluation and ranking of membership functions of 18 *E. sibiricus* samples.

Materials	OPC	FLA	Chl	RWC	EL	SP	PRO	POD	SOD	Average value	Order
SC020 2-A1	0.64	0.51	0.78	0.77	0.62	0.48	0.62	0.63	0.81	0.65	1
SC021 12-A2	0.56	0.44	0.67	0.80	0.55	0.54	0.64	0.68	0.54	0.60	2
Mai-wa	0.57	0.45	0.71	0.79	0.62	0.50	0.50	0.56	0.71	0.60	3
QH010 41-A1	0.44	0.62	0.64	0.78	0.59	0.44	0.51	0.63	0.61	0.58	4
NM050 37-A13	0.56	0.48	0.51	0.57	0.55	0.45	0.42	0.47	0.52	0.50	5
GS017 5-A2	0.52	0.36	0.54	0.63	0.56	0.40	0.54	0.47	0.47	0.50	6
XJ021 29-A8	0.35	0.45	0.50	0.60	0.43	0.36	0.49	0.49	0.67	0.48	7
SC017 11-A4	0.36	0.41	0.55	0.57	0.49	0.33	0.55	0.52	0.51	0.48	8
QH016 26-A9	0.32	0.41	0.38	0.64	0.58	0.38	0.29	0.52	0.62	0.46	9
QH009 33-A9	0.35	0.43	0.41	0.67	0.42	0.37	0.59	0.43	0.46	0.46	10
NM044 30-A13	0.52	0.27	0.57	0.66	0.50	0.35	0.37	0.32	0.51	0.45	11
XJ001 30-A8	0.54	0.40	0.45	0.59	0.35	0.39	0.37	0.55	0.30	0.44	12
XZ015 22-A6	0.52	0.25	0.48	0.46	0.36	0.32	0.38	0.36	0.41	0.39	13
GS009 10-A8	0.30	0.59	0.28	0.36	0.32	0.29	0.31	0.41	0.49	0.37	14
XZ008 9-A2	0.41	0.28	0.54	0.21	0.51	0.42	0.24	0.32	0.42	0.37	15
SC011 8-A2	0.38	0.35	0.41	0.42	0.31	0.36	0.38	0.25	0.38	0.36	16
HB005 13-A3	0.26	0.35	0.32	0.32	0.30	0.45	0.36	0.34	0.30	0.33	17
XJ007 22-A5	0.30	0.26	0.21	0.41	0.23	0.37	0.35	0.35	0.33	0.31	18

Table S2. Morphological profiles of UV-B tolerant (SC) and UV-B sensitive (XJ) *E. sibiricus* genotypes.

Index	Code	UV-B radiation stress gradient				
		0 d	5 d	10 d	15 d	20 d
Blade damage level	SC	0	0	1	2	3
	XJ	0	1	2	3	4

Note: 0, 1, 2, 3, and 4 respectively denote leaf greenness, marginal chlorosis, leaf wilting, curling necrotic spots, and plant withering.

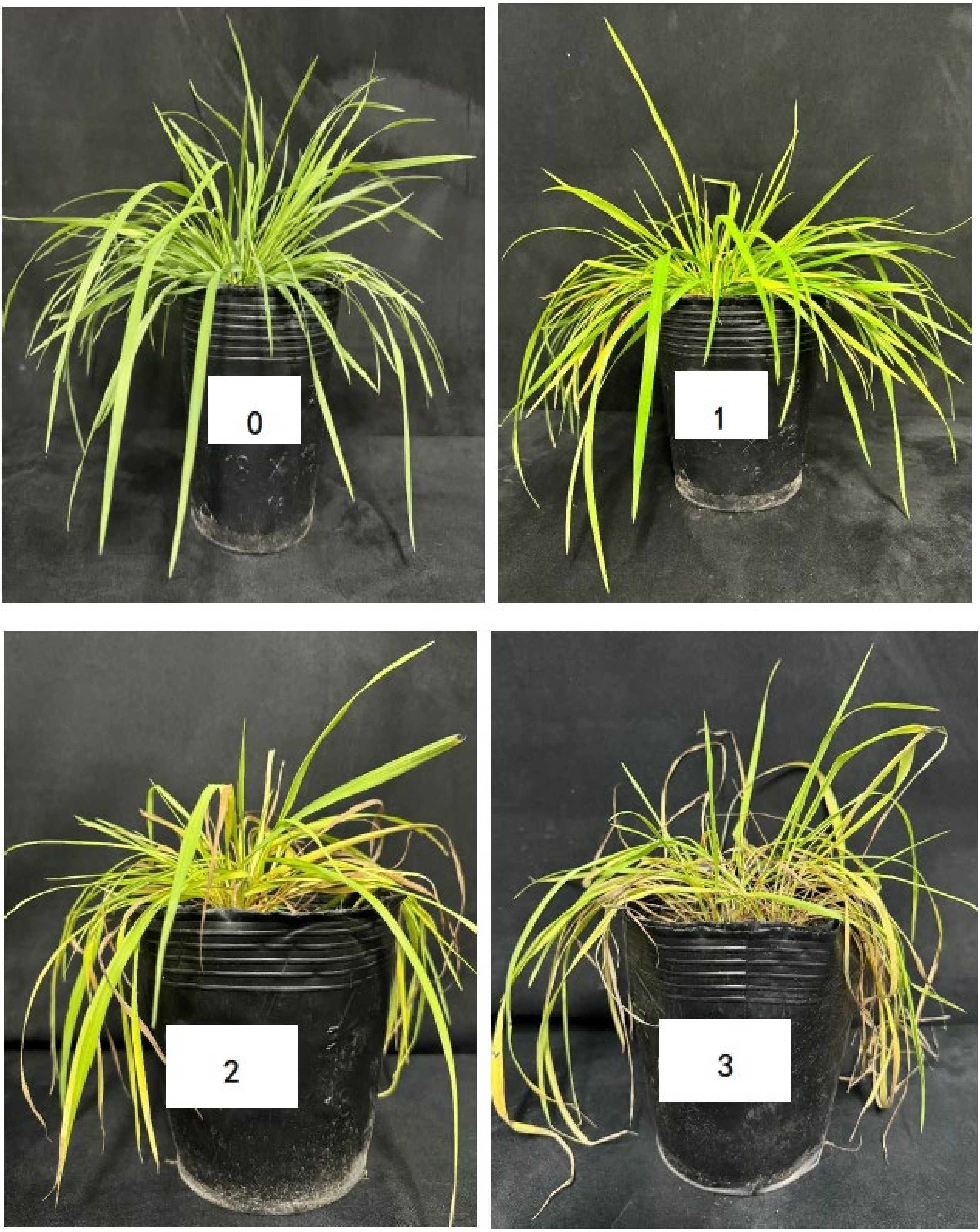
0
1
2
3

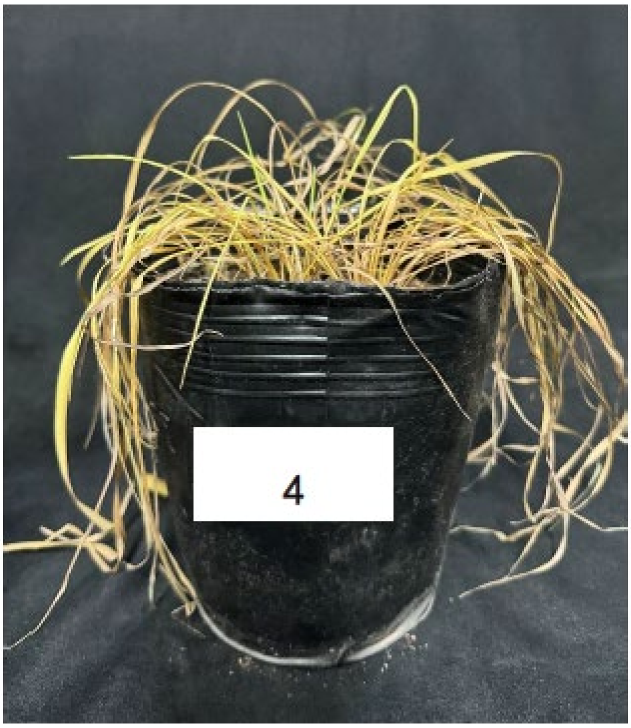
4
